# Cognitive reserve and mental health in cognitive frailty phenotypes: Insights from a study with a Portuguese sample

**DOI:** 10.3389/fpsyg.2022.968343

**Published:** 2022-08-30

**Authors:** Pedro Miguel Gaspar, María Campos-Magdaleno, Arturo X. Pereiro, David Facal, Onésimo Juncos-Rabadán

**Affiliations:** ^1^UNICES, Universidade da Maia, Maia, Portugal; ^2^Department of Developmental Psychology, University of Santiago de Compostela, Santiago de Compostela, Spain

**Keywords:** subjective cognitive decline, mild cognitive impairment, education, social-support, leisure activities, affective disorders

## Abstract

**Background:**

Research on prevalence of cognitive frailty phenotypes in community-dwelling older adults in different countries is important to estimate their prevalence and to determine the influence of cognitive reserve and mental health in order to prevent frailty. The aims of this study were to estimate the prevalence of reversible and potentially reversible cognitive frailty (R-CF, PR-CF) in a Portuguese sample of old adults and explore the associations between these phenotypes and demographic, comorbidity, social support, cognitive reserve and mental health factors.

**Methods:**

We assessed frailty (Fried criteria) in 250 community-dwelling older adults (179 women) aged 60 years or over (mean 71.04 years) without dementia, neurological or psychiatric disorders. Subjective cognitive decline and Mild cognitive impairment were diagnosed according to standard criteria. The questionnaires Charlson Index, Medical Outcomes Study Social Support, Cognitive Reserve Index and General Health were used for assessing comorbidity, social support, cognitive reserve and mental health, respectively.

**Results:**

Prevalence of R-CF was 14%, and that of PR-CF, 15.2%. Cognitive frailty profiles differed significantly in relation to education, comorbidity, mental health, and cognitive reserve, but not in age or sex. Multivariate logistic regression showed that age, sex, comorbidity, social support, mental health, and cognitive reserve together predicted R-CF and PR-CF (90% specificity 75% sensitivity) with significant OR for mental health and cognitive reserve.

**Discussion:**

Cognitive reserve and mental health are important factors predicting R-CF and PR-CF. We recommend assessing these factors for early detection of cognitive frailty and promoting psychological well-being and lifestyles that increase cognitive reserve in adults.

## Introduction

Cognitive frailty (CF) is currently defined as the simultaneous presence of physical frailty and cognitive impairment (CDR = 0.5) with the exclusion of concurrent AD dementia or other types of dementia (Kelaiditi et al., 2013). A new definition of CF as a heterogeneous syndrome of cognitive impairment (CDR ≤ 0.5) caused by physical frailty or physical pre-frailty, but excluding all types of dementia, has recently been proposed (Ruan et al., 2015). This broader concept includes two subtypes, reversible cognitive frailty (R-CF) and potentially reversible cognitive frailty (PR-CF), which correspond, respectively, to two levels of cognitive impairment, i.e., subjective cognitive decline (SCD; Jessen et al., 2014) and mild cognitive impairment (MCI), which represent different stages in the continuum of cognitive decline (Jack et al., 2018).

Community-based studies have shown the prevalence of PR-CF in older adults to vary from 4.4% (Roppolo et al., 2017) to 6.3% (Ruan et al., 2020) considering only physical frailty, or 21.8% considering physical frailty and physical pre-frailty (Navarro-Pardo et al., 2020). These different rates may be explained by differences in the way the CF components operate and are measured (Facal et al., 2019). Studies on the prevalence of R-CF are scarce, but it has been suggested that the prevalence can reach up to 19.8% (Ruan et al., 2020). PR-CF has been associated with increasing age, and R-CF has been associated with age, sex, and education (Ruan et al., 2020).

Although cognitive reserve (CR) is one of the parameters used to characterize CF (Kelaiditi et al., 2013) the relationship between CR and frailty represents a very recent research issue. CR refers to the efficiency, capacity and flexibility of cognitive processes that explain differential of brain damage in cognitive performance and day-to-day functioning (Stern, 2009). It is an active model of resilience influenced by different exposures across the lifespan, including but not limited to early general cognitive ability (intelligence), formal education, occupation, physical exercise, leisure activities and social participation (Livingston et al., 2020; Stern et al., 2020). Several published studies suggest a potential protective role of cognitive reserve factors against the onset and worsening of frailty among older adults, keeping their cognitive abilities at older ages (Staff et al., 2004). A recent review highlighted the fact that the only proxy for CR included in studies of CF is the level of formal education and that a low level has been indicated as a risk factor for CF (Facal et al., 2021). People with a lower educational level are more exposed to cognitive frailty at later age (Gutierrez-Robledo and Avila-Funes, 2012; Gale et al., 2020). However, the relationship between other CR dimensions, apart from years of education, as profession, social, cultural, family, and free-time activities throughout life (Stern, 2009), and CF has not yet been clearly established. There are also few studies addressing the association between proxies for CR and physical frailty (Sardella et al., 2020).

Psychological disturbances such as stress and depression are determining factors and essential components that can contribute to frailty (Bilotta, 2010; Kuiper et al., 2015). The assessment of mental health and affective disorders that impair psychological well-being has been pointed out as an important procedure in the early detection of frailty (Andrew and Rockwood, 2007; Lin et al., 2020). Mental health is associated with an increase prevalence of frailty and may be a risk factor for its development in older adults (Soysal et al., 2017). Recent studies found also significant specific association between CF and mental health status (Navarro-Pardo et al., 2020; Xie et al., 2021).

Portugal is one of the European countries with an older population; life expectancy at birth will significantly increase from 82 to 87 years between 2019 and 2070, and the rate of older adults (65 and over) will be of 24.2% in 2025 and 30.4% in 2050 (European Commission, Directorate-General for Economic and Financial Affairs, 2021). Portugal was the third country with higher prevalence of physical pre-frailty (47.6%) and the first with physical frailty (15.6%) of the 18 European countries included in the SHARE study (Manfredi et al., 2019). Moreover, the Portuguese older adults have particular sociocultural characteristics characterized by a low average education with predominance of the 1st cycle (primary education) or without schooling (Moreira, 2020) and a high level of early school leaving for historical reasons of socio-economic nature (Gonçalves et al., 2021). At our knowledge there is not any study in Portugal devoted to analyze different cognitive frailty phenotypes and their associated factors such as cognitive reserve and mental health that are important to prevent cognitive frailty (Facal et al., 2021).

In the current research we studied cognitive frailty phenotypes in a Portuguese sample of community-dwelling older adults. We assessed, for the first time, the prevalence of the phenotypes in the Portuguese older adults, and we analyzed the associations between these phenotypes and age, sex, comorbidity, social support, and mental health. We studied with special interest the association between cognitive frailty phenotypes and the cognitive reserve dimensions included in the Stern model (Stern, 2009), i.e., education, profession, and social, cultural, family and free-time activities throughout life.

## Materials and methods

### Participants

This population-based study included an incidental sample comprised of 250 participants (179 women, 71 men) of mean age 71.04 years (SD = 8.18) and with 6.56 years of mean of formal education (SD = 4.17; Table 1). Observing the general Portuguese demographics, our sample is very balanced in view of distribution by the considered age groups (Data Sources: INE, 2021). The participants were recruited in 2019 through relatives or friends of older adults attending the “Hospital da Prelada, Misericordia do Porto” in the District of Porto (north-west Portugal). All participants met the following criteria: aged 60 years or more, without prior diagnosis of dementia or neurological or psychiatric disorders; without traumatic injuries or sensory or motor deficiencies that prevent evaluation; without serious gait disturbance or technical assistance; and without dependency for instrumental activities of daily life. The “Misericordia do Porto” Ethics Committee approved the study, in accordance with the Declaration of Helsinki updated in Fortaleza (World Medical Association Declaration of Helsinki, 2013), and each participant gave informed consent to participate in the study.

**Table 1:** Socio-demographic, cognitive and physical profiles.

Variables	Cognitive profiles (all sample)					
	G1. Cognitively unimpaired (*n* = 125; 50%)	G2. Subjective cognitive decline (*n* = 49; 19.6%)	G3. Mild cognitive impairment (*n* = 49; 19.6%)	G4. Moderate cognitive impairment (*n* = 27; *n* = 10.8%)	Total (*N* = 250; 100%)	*H* de Kruskal-Wallis; Groups comparisons
Age	70.37 (7.37)	69.92 (7.15)	71.24 (9.64)	75.85 (9.37)	71.04 (8.18)	8.12^*^ G4 > G1, G2, G3
Sex	88 women/37 men	31 women/18 men	35 women/14 men	25 women/2 men	179 women/71 men	7.61 NS
Formal education (years)	6.23 (3.70)	8.35 (5.32)	5.73 (3.27)	6.30 (4.58)	6.56 (4.17)	7.23 NS
Charlson index	0.42 (0.59)	0.37 (0.63)	0.78 (1.27)	0.96 (0.64)	0.54 (0.81)	19.89^**^ G4 > G1, G2, G3
EMQ-11	5.13 (4.06)	14.85 (4.03)	8.34 (6.18)	16.92 (6.98)	8.94 (6.70)	108.98^**^ G4 > G2, G3, G1 G2 > G1, G3
MoCA	23.83 (3.47)	25.02 (3.56)	17.57 (3.14)	10.63 (4.78)	21.41 (5.81)	133.07^**^ G1, G2 > G3, G4
MOSS-SS	84.78 (15.45)	83.35 (12.73)	82.73 (17.75)	79.15 (16.59)	83.49 (15.57)	4.53 NS
GHQ-12	8.57 (4.22)	11.20 (5.61)	10.93 (6.24)	18.74 (8.57)	10.65 (6.28)	41.02^**^ G4 > G1, G2, G3 G2 > G1
CRIq-Total	94.61 (14.70)	98.71 (14.81)	87.63 (12.50)	81.70 (14.64)	92.65 (15.15)	34.76^**^ G1, G2 > G3 > G4
CRIq-Education	89.66 (10.40)	94.10 (13.16)	88.96 (9.04)	94.00 (13.54)	90.86 (11.26)	5.27 NS
CRIq-Working activity	102.18 (13.09)	105.49 (15.61)	97.49 (11.78)	94.89 (11.32)	101.12 (13.55)	13.44^**^ G1, G2 > G3, G4
CRIq-Leisure time	98.98 (19.66)	97.59 (15.13)	85.59 (17.96)	70.04 (16.70)	91.46 (20.05)	45.98^**^ G1, G2 > G3 > G4
**Physical profiles**						
		**G1. Robust (*n* = 68; 27.2%)**	**G2. Pre-frail (*n* = 108; 43.2%)**	**G3. Frail (*n* = 74; 29.6%)**		
Age		68.37 (6.84)	70.15 (8.01)	74.81 (8.31)		23.89^**^ G3 > G1, G2
Sex		45 women/23 men	80 women/28 men	54 women/20 men		1.37 NS
Formal education (years)		7.82 (4.69)	6.77 (4.04)	5.08 (3.37)		20.65^**^ G3 < G1, G2
Charlson index		0.28 (0.54)	0.44 (0.58)	0.93 (1.11)		26.24^**^ G3 > G1, G2
EMQ-11		7.02 (6.47)	7.56 (5.45)	12.71 (7.11)		31.17^**^ G3 > G1, G2
MoCA		24.15 (4.18)	22.43 (4.25)	17.42 (6.92)		41.96^**^ G3 < G1, G2
MOSS-SS		88.93 (11.71)	82.69 (16.33)	79.65 (16.34)		15.97^**^ G1 > G2, G3
GHQ-12		6.85 (2.01)	9.28 (4.31)	16.13 (7.56)		81.45^**^ G3 > G2 > G1
CRIq-Total		97.50 (16.36)	94.32 (13.32)	85.76 (14.24)		27.14^**^ G1 > G2 > G3
CRIq-Education		91.74 (12.06)	90.73 (11.48)	90.24 (10.23)		0.34 NS
CRIq-Working activity		103.04 (13.66)	101.47 (13.78)	98.85 (12.96)		2.05 NS
CRIq-Leisure time		99.69 (20.25)	94.95 (17.79)	78.80 (16.97)		42.61^**^ G1 > G2 > G3

Descriptive parameters (mean, standard deviation in parentheses, frequency) and groups comparisons. EMQ-11, Everyday Memory Questionnaire; MoCA, Montreal Cognitive Assessment; MOS-SS, Medical Outcomes Study Social Support Survey; GHQ-12, General Health Questionnaire; CRIq, Cognitive Reserve Index Questionnaire.

* *p* < 0.05;

** *p* < 0.01.

### Measurement

The measurement protocol included a socio-demographic questionnaire and physical and cognitive questionnaires or tests to evaluate and define physical and cognitive frailty. Comorbidity was assessed with the Charlson Comorbidity Index (Charlson et al., 1987). Social support was assessed with the Portuguese version of Medical Outcomes Study Social Support questionnaire (MOS-SS; Sherbourne and Stewart, 1991; Fachado et al., 2007) that evaluates functional support in four dimensions, material support, emotional support, affective support, and positive social interaction. The total reliability of the Portuguese version of MOS-SS is Cronbach’s alpha = 0.97. Higher scores indicate greater social support. Mental health was evaluated with the Portuguese version of General Health Questionnaire (GHQ-12; Goldberg, 1992; Goldberg et al., 1997; Borges and Argolo, 2002; total reliability, Cronbach alpha = 0.88). It assesses clinical non-psychotic psychiatric disorders, and higher scores indicate greater probability of suffering them. Cognitive reserve was assessed with the Cognitive Reserve Index questionnaire (CRIq; Nucci et al., 2012, Portuguese version) which yields a total score and partial scores for three dimensions CRIq Education, CRIq Working activity, and CRIq Leisure time, including, respectively, schooling, profession, and social, cultural, family and free-time activities throughout life. The total reliability of CRIq is Cronbach’s alpha = 0.62 (95% CI 0.56–0.97) and for the dimension CRIq Leisure time is Cronbach’s alpha = 0.73 (95% CI 0.70–0.76).

### Definition of physical frailty

The following five criteria (Fried et al., 2001) were used to assess frailty phenotypes: (1) self-reported exhaustion or fatigue; (2) weakness, measured by the grip strength of the dominant hand with a calibrated dynamometer and using the mean value of three measurements (cut-off <26 for men and < 16 for women; Alley et al., 2014); (3) slow ambulation measured in a timed-up and go task (Podsiadlo and Richardson, 1991; Dutra et al., 2016) in which the participant has to get up from a chair, walk a distance of 3 m, turn around, walk back towards the chair, and sit down again, with a cut-off of >20 s; (4) low level of physical activity or sedentarism, measured with a short version of Minnesota leisure time Physical Activity Questionnaire (Taylor et al., 1978); and (5) weight loss, measured with yes/ no responses about unintentional weight loss and lack of appetite in the last 3 months. Participants were classified into three frailty phenotypes, depending on the number of criteria fulfilled: non-frail/robust (none of the criteria fulfilled); pre-frail (one or two criteria); and frail (three to five criteria).

### Definition of reversible cognitive frailty

Reversible cognitive frailty was defined by the simultaneous presence of physical frailty and physical pre-frailty (Fried et al., 2001) and SCD. Participants were diagnosed as SCD when they met the two main criteria proposed by the SCD-initiative (SCD-I) Working Group (Jessen et al., 2020): (1) self-experienced persistent decline in cognitive capacity, especially in memory, relative to a previously normal cognitive status, which is unrelated to an acute event; and (2) normal performance in standardized cognitive tests used to classify MCI, adjusted for age, sex and education. To determine the first criterion, we asked the participants if they were worried about their failures in attention and memory in the last few years. We used the Everyday Memory Questionnaire (EMQ), short version of 11 items (Sunderland et al., 1984; Ávila-Villanueva et al., 2016) which includes questions on memory and other cognitive domains, such as perception, attention language and executive functions, to determine whether their cognitive failures were worse than those of other people of the same age. For this purpose, we established cut-off scores as the upper limit of the confidence interval of the mean scores according to the age groups (60–69, 70–79, 80+ years) in the sample. Normal cognitive performance was assessed with the Portuguese version of the Montreal Cognitive Assessment (MoCA) test (Nasreddine et al., 2005; Freitas et al., 2011) with norms for age and education. Three groups of participants were established according to these criteria: no physical frailty and SCD, physical pre-frailty and SCD, and physical frailty and SCD. The two last groups were diagnosed as having reversible cognitive frailty.

### Definition of potentially reversible cognitive frailty

Potentially reversible cognitive frailty was defined by the simultaneous presence of physical frailty (pre-frail and frail) and MCI. MCI was diagnosed according to the criteria established by Albert et al. (2011). The cut-off scores for cognitive impairment were performance levels of 1–2 standard deviations below the MoCA Portuguese norms for age groups (60–69, 70–79, 80+ years) and education levels (0–4, 5–9, 10–12, 13+ years of formal education; Gonçalves et al., 2021). Participants with MoCA scores below 2 Standard deviations and who did not meet the criteria for dementia (DSM-5) were categorized as having moderate cognitive impairment (ModCI) and were not included into the cognitive frailty phenotypes. Participants were classified into three groups on the basis of physical frailty and MCI diagnoses: no physical frailty and MCI, physical pre-frailty and MCI, and physical frailty and MCI. The last two groups were considered to have potentially reversible cognitive frailty.

### Statistical analyses

All statistical analyses were performed with SPSS for Windows, version 26.0 (IBM corp. Armonk, NY, United States) using a cut-off *p* value of 0.05. Frequencies and percentages were calculated for categorical variables. As the variances were not homogeneous, we used nonparametric Kruskal–Wallis and Mann–Whitney tests to compare the different profiles in relation to all of the variables considered. We used multivariate logistic regression models to determine the odds ratios with 95% confidence intervals. Participants with reversible cognitive frailty and physical frailty (pre-frail and frail) and SCD were classed as cases and participants without physical frailty and cognitively unimpaired as controls; for potentially cognitive frailty, participants with physical frailty (pre-frail and frail) and MCI were classed as cases, and participants without physical frailty and cognitively unimpaired were classed as controls. We examined two different models with independent variables or covariates in order to test the alternative value of years of education against the three dimensions of cognitive reserve: Model A in which the covariates were age groups (60–69, 70–79, 80+ years), sex, comorbidity (Charlson index), social support (MOSS-SS, total score), mental health (GHQ-12, total score) and the three dimensions of cognitive reserve (CRIq Education, CRIq Working activity and CRIq Leisure time); and Model B, in which the cognitive reserve covariates were replaced with years of formal education.

## Results

The total sample was of mean age 71.04 years (SD 8.18), with a mean of 6.52 (SD 4.17) of years of formal education. Of the 250 participants, 71% were women. Regarding cognitive status, 50% of participants were categorized as cognitively unimpaired (CU), 19.6% as having SCD, 19.6% as having MCI and 10.8% as having moderate cognitive impairment (ModCI). Regarding physical status, 27.2% were categorized as robust (16.8% CU, 5.6% SDC, 4.4% MCI and 0.4% ModCI) 43.2% as pre-frail (24.8% CU, 8.4% SDC, 7.6% MCI and 2.4% ModCI) and 29.6% as frail (8.4% CU, 5.6% SCD, 7.6% MCI and 8% ModCI). The characteristics of the total sample and of the corresponding cognitive and physical profiles are shown in Table 1. The prevalence of reversible cognitive frailty was 14% and that of potentially reversible cognitive frailty, 15.2% (see Table 2).

**Table 2:** Cognitive frailty profiles.

Variables	Cognitive frailty profiles					
	Reversible cognitive frailty		Potentially reversible cognitive frailty		Total sample (*N* = 250 100%)	
	G1 Pre-frail + SCD (*n* = 21; 8.4%)	G2 Frail + SCD (*n* = 14; 5.6%)	G3 Pre-frail + MCI (*n* = 19; 7.6%)	G4 Frail + MCI (*n* = 19; 7.6%)	Total (*n* = 73)	*H* de Kruskal-Wallis; Groups comparisons
Age	69.10 (6.75)	73.14 (8.05)	70.05 (10.41)	75.11 (9.51)	71.68 (8.196)	6.83 NS
Sex	14 women/7 men	8 women/6 men	13 women/6 men	14 women/5 men	49 women/24 men	1.01 NS
Formal education (years)	9.86 (5.03)	5.36 (2.95)	6.89 (3.79)	3.84 (1.70)	6.66 (4.28)	21.22^**^ G1 > G2,G3 > G4
Charlson index	0.33 (0.65)	0.64 (0.63)	0.53 (0.69)	1.27 (1.77)	0.71 (1.12)	8.54^*^ G1 < G4
EMQ-11	13.95 (3.68)	14.07 (3.56)	6.31 (4.74)	12.21 (6.70)	11.53 (5.76)	20.47^**^ G1,G2,G4 < G3
MoCA	25.76 (2.68)	22.71 (4.21)	18.84 (2.69)	15.32 (2.79)	20.66 (5.07)	47.60^**^ G1 > G2 > G3 > G4
MOS-SS	79.76 (15.37)	82.86 (11.66)	81.63 (20.40)	78.21 (17.85)	80.44 (16.64)	1.37 NS
GHQ-12	11.00 (4.65)	15.42 (6.81)	9.47 (5.45)	14.47 (7.05)	12.35 (6.32)	11.27^*^ G1,G3 < G2,G4
CRIq-Total	102.90 (13.63)	92.86 (13.41)	91.37 (14.64)	82.11 (9.75)	92.56 (14.89)	22.70^**^ G1 > G2,G3 > G4
CRIq-Education	97.05 (11.85)	89.64 (10.48)	90.53 (11.29)	87.79 (7.20)	91.52 (10.83)	6.34 NS
CRIq-Working activity	107.76 (17.66)	101.29 (13.02)	98.00 (14.97)	95.95 (10:05)	100.90 (14.86)	4.72 NS
CRIq-Leisure time	102.00 (14.47)	92.71 (15.60)	91.95 (19.34)	75.74 (13.70)	90.77 (18.44)	20.49^**^ G1,G2,G3 > G4

Descriptive parameters (mean, standard deviation in parentheses, frequency) and groups comparisons. EMQ-11, Everyday Memory Questionnaire; MoCA, Montreal Cognitive Assessment; MOS-SS, Medical Outcomes Study Social Support Survey; GHQ-12, General Health Questionnaire; CRIq, Cognitive Reserve Index Questionnaire.

* *p* < 0.05; and

** *p* < 0.01.

Comparison of the cognitive profiles showed that participants with ModCI were older, with higher comorbidity (Charlson Comorbidity Index) and poorer mental health (GHQ-12) than the cognitively unimpaired, SCD and MCI participants. Cognitive reserve (CRIq Total, CRIq Working activity and CRIq Leisure time) was higher in the CU and SCD participants than in MCI and ModCI participants. The groups did not differ significantly in relation to sex, education (formal education and CRIq Education) or social support (MOSS-SS; Table 1). Regarding physical profiles, the frail participants were older, with fewer years of formal education, higher comorbidity, more subjective cognitive complaints (EMQ) and poorer objective cognitive performance (MoCA) than robust and pre-frail participants; social support was higher for robust than for pre-frail and frail participants; mental health was poorer for the pre-frail and frail than for the robust participants; cognitive reserve (CRIq Total and CRIq Leisure time) was higher for robust than for pre-frail and frail participants, and it was also higher for pre-frail than for frail participants. There were no significant differences between the groups in relation to sex, CRIq education or CRIq Working activity (Table 1).

The cognitive frailty profiles showed significant differences in formal education, comorbidity, mental health and cognitive reserve (Table 2). The Pre-frail + SCD group had more years of education than the other three groups, and the Frail + SCD and Pre-frail + MCI more than the Frail + MCI. Comorbidity (Charlson Comorbidity Index) was higher only in the Frail + MCI group relative to the Pre-frail + SCD group. The probability of mental clinical disorders (GHQ-12) was higher in the Frail + SCD and Frail + MCI groups than in Pre-frail + SCD and Pre-frail + MCI groups. Cognitive reserve (CRIq Total) was higher in the Pre-Frail + SCD group than in the other three groups and higher in the Frail + SCD and Pre-frail +MCI groups than in the Frail + MCI group; this group also obtained lower scores in CRIq Leisure time than the other three groups.

Multivariate logistic regression analysis showed that the model with the covariates age (age group), sex, comorbidity (Charlson Comorbidity Index), social support (MOSS-SS), mental health (GHQ-12) and cognitive reserve (CRIq education, CRIq working activity and CRIq leisure) predicted Reversible cognitive frailty with a good fit index (*χ*^2^ Hosmer and Lemehow = 8.443, *p* = 0.391), 90.5% specificity, 74.3% sensitivity, and explained 69.2% of the variance (*R*^2^ of Nagelkerke = 0.692). Mental health (GHQ-12) was the only covariate with a significant OR (OR = 2.257; 95% CI = 1.393–3.658; Table 3, Model 1). We also tested another model, in which the CR variables were replaced with Years of formal education, and which also yielded a good fit index (*χ*^2^ Hosmer and Lemehow = 9.167, *p* = 0.328), with 90.5% specificity, 74.3% sensitivity, and 66% of the variance explained (*R*^2^ of Nagelkerke = 0.660), with GHQ-12 as the only variable with significant OR (OR = 2.164; 95% CI = 1.403–3.337; Table 3, Model 2). The same covariates were included in the model predicting Potentially reversible cognitive frailty (Table 4, Model 3), yielding a good fit (*χ*^2^ Hosmer and Lemehow = 9.643, *p* = 0.291), with 90.5% specificity, 76.3% sensitivity and 58.3% of the variance explained (*R*^2^ of Nagelkerke = 0.583). In this case the significant variables were GHQ-12 (OR = 1.722; 95% CI = 0.210–2.450) and CRIq Leisure time (OR = 0.961; 95% CI = 0.924–0.999). The alternative model (Table 4, Model 4) including years of education instead of cognitive reserve also yielded a good fit (*χ*^2^ Hosmer and Lemehow = 3.826, *p* = 0.872), but lower specificity (83.3%) and sensitivity (68.4%), with 50.5.% of the variance explained (*R*^2^ of Nagelkerke = 0.550), and GHQ-12 the only variable with significant OR (OR = 1.559; 95% CI = 1.170–2.076).

**Table 3:** Multivariate regression analysis.

**Covariate**	***R***^**2**^	***B***	**SE**	**Wald**	***p*-value**	**OR**	**95% CI**
**(A) Model 1**							
	0.692						
Age group (years)							
60–69						1	
70–79		−0.944	0.872	1.117	0.279	0.389	0.070–2.151
80+		0.666	1.098	0.368	0.544	1.947	0.226–16.749
Sex							
Women						1	
Men		0.264	0.808	0.107	0.743	1.303	0.267–6.345
Charlson comorbidity index		0.742	0.673	1.217	0.270	2.101	0.562–7.853
MOSS-SS		−0.025	0.024	1.135	0.287	0.975	0.931–1.021
GHQ-12		0.814	0.246	10.920	0.001	2.257	1.393–3.658
CRIq-Education		0.062	0.049	1.649	0.199	1.064	0.968–1.171
CRIq-Working activity		0.021	0.036	0.337	0.561	1.021	0.951–1.096
CRIq-Leisure time		−0.036	0.020	3.158	0.076	0.964	0.927–1.004
**(B) Model 2**							
	0.660						
Age group (years)							
60–69						1	
70–79		−0.432	0.780	0.307	0.580	0.649	0.141–2.997
80+		1.775	1.108	2.564	0.109	5.899	0.672–51.780
Sex							
Women						1	
Men		−0.334	0.768	0.189	0.664	0.716	0.159–3.230
Charlson comorbidity index		0.992	0.623	2.192	0.139	2.515	0.742–8.527
MOSS-SS		−0.029	0.022	1.754	0.185	0.972	0.931–1.014
GHQ-12		0.772	0.221	12.174	0.001	2.164	1.403–3.340
Years of education		0.127	0.080	2.480	0.115	1.135	0.969–1.329

Reversible cognitive frailty. MOS-SS: Medical Outcomes Study Social Support Survey; GHQ-12: General Health Questionnaire; CRIq: Cognitive Reserve Index Questionnaire.

**Table 4:** Multivariate regression analysis.

Covariate	*R* ^2^	*B*	SE	Wald	*p*-value	OR	95% CI
**(A) Model 3**							
	0.583						
Age group (years)							
60–69						1	
70–79		−0.748	0.936	0.639	0.424	0.424	0.076–2.963
80+		0.101	1.001	0.010	0.919	1.107	0.156–7.872
							
Sex							
Women						1	
Men		−0.542	0.744	0.530	0.467	0.582	0.135–2.501
							
Charlson Comorbidity Index		0.595	0.501	1.410	0.235	1.812	0.679–4.835
							
MOS-SS		−0.019	0.022	0.793	0.373	0.981	0.940–1.024
							
GHQ-12		0.543	0.180	9.120	0.003	1.722	1.210–2.450
							
CRIq-Education		0.019	0.044	0.191	0.662	1.019	0.936–1.111
CRIq-Working Activity		−0.027	0.034	0.652	0.419	0.963	0.910–1.040
CRIq-Leisure Time		−0.040	0.020	4.093	0.043	0.961	0.924–0.999
**(B) Model 4**							
	0.505						
Age group (years)							
60–69						1	
70–79		−0.769	0.785	0.961	0.327	0.463	1.000–2.157
80+		0.500	0.771	0.420	0.515	1.649	0.364–7.478
Sex							
Women						1	
Men		0.498	0.666	0.558	0.455	1.645	0.446–6.066
Charlson Comorbidity Index		0.728	0.477	2.327	0.127	2.072	0.813–5.281
MOS-SS		−0.018	0.019	0.861	0.353	0.982	0.946–1.020
GHQ-12		0.444	0.146	9.206	0.002	1.559	1.170–2.076
Years of Education		−0.093	0.080	1.358	0.244	0.911	0.778–1.111

Potentially reversible cognitive frailty. MOS-SS: Medical Outcomes Study Social Support Survey; GHQ-12: General Health Questionnaire; CRIq: Cognitive Reserve Index Questionnaire.

## Discussion

The observed rates of physical pre-frailty and physical frailty (43.2% and 29.6%) were quite similar to those reported in other studies that used similar assessment procedures (Roppolo et al., 2017; Navarro-Pardo et al., 2020) taking into account that in the present study, participants with moderate cognitive impairment were considered in determining physical frailty. Comparing our results with those reported by Manfredi et al. (2019) from the SHARE study for Portugal we observe a similar rate for physical pre-frailty (43.2% versus 47.6%) whereas our incidence of physical frailty was higher (29.6%) than that of the SHARE study (15.6%). Difference in physical frailty rates could be due to the different criterion used to evaluate slowness. We used a more objective criterion based on performance in the timed-up and go test (Podsiadlo and Richardson, 1991) whereas Manfredi et al. (2019) used the participants’ responses to mobility questions (such as “Please tell me whether you have any difficulty for climbing one flight of stairs without resting” or “for walking 100 m”).

The prevalence of reversible and potentially cognitive frailty was 14% and 15.2%, respectively. Our rate of reversible cognitive frailty was lower than that reported by Ruan and colleagues (19.86%; Ruan et al., 2020). This difference may be due to the different procedures used to diagnose SCD. These authors used a positive response to 1 of 2 questions as a criterion for diagnosing SCD (“In the last 2 years, has your memory declined?” or “Has your other cognition declined, such as having difficulty remembering family members’ or close friends’ names, finding your way around your neighbourhood, or handling money?”). We think that our psychometric determination of poorer subjective cognitive status, relative to other people of the same age group (determined from the upper confidence intervals) is probably a more accurate way of diagnosing SCD. The rate of potentially reversible cognitive frailty (15.2%) was higher than the 6.3% reported by Ruan et al. (2020) and closer to the 21.8% reported by Navarro-Pardo et al. (2020). The cultural and socio-demographic similarities between the older Portuguese population and the Spanish population studied by Navarro-Pardo et al. (2020) together with the similar methodology for determining physical frailty (Fried et al., 2001) and MCI (by the MoCA test) may explain the similar rates in the two studies. Conversely the different rates obtained by Ruan and colleagues may be due, on the one hand, to the use of the Rapid Cognitive Screen (Malmstrom et al., 2015) for assessing cognitive dysfunction, as this test displays weak sensitivity (0.62%) and specificity (0.62%) for MCI and, on the other hand, to the cultural and socio-demographic characteristics of the Chinese sample.

Our comparison of the physical profiles, showed the following: (a) the already established role of old age, low education level and higher comorbidity in differentiating frailty from pre-frailty (Merchant et al., 2017; Brigola et al., 2019); (b) the lower subjective and objective cognitive status of frailty (Brigola et al., 2015); (c) the importance of other psychosocial variables such as social support and mental health in characterizing frailty (Andrew and Rockwood, 2007; Facal et al., 2019); (d) the potentially protective value (against frailty) of cognitive reserve, mainly social, cultural and leisure activities developed throughout life (Sardella et al., 2020); and (e) the lack of sex-related differences.

Comparison of the cognitive frailty profiles not only confirmed that low level of education differentiates the frailest group (Frail + MCI) from the others (Niederstrasser et al., 2019), but it also demonstrated the differentiating role of cognitive reserve and mainly of the Leisure activities component. Comorbidity was associated with physical frailty (Fried et al., 2001) and with the combined presence of physical frailty and MCI (Garre-Olmo et al., 2013) The probability of mental clinical disorders was higher in both frailty profiles (with SCD and with MCI) than in the two pre-frailty profiles. This finding underlines the importance of including mental health as a psychosocial factor in a broader and integral model of frailty (Gobbens et al., 2010; Panza et al., 2018; Navarro-Pardo et al., 2020). No significant differences were found in relation to age or sex.

Our findings from multivariate logistic regression analyses show the importance of the joint consideration of the socio-demographic variables age, sex, years of formal education, comorbidity (as a proxy for physical heath), the psychosocial variables social support and (particularly) mental health, and cognitive reserve (particularly social, cultural and leisure activities) for predicting reversible and potentially reversible cognitive frailty. Our model correctly classified 90.5% of physically robust and cognitively unimpaired control participants, and 74.3% of case participants with reversible cognitive frailty (Table 3, Model 1). Regarding potentially reversible cognitive frailty, the model correctly classified 90.5% of physically robust control participants and 76.3% of case participants with potentially reversible cognitive frailty (Table 4, Model 3). Although all the variables included in the models contribute to the good fit and good parameters related to explanation of variance, specificity and sensitivity, in the models including reversible cognitive frailty, only the variable GHQ-12 (mental health) produced a significant OR, and in the models examining potentially reversible cognitive frailty the only variables with significant OR were GHQ-12 and Cognitive reserve leisure activities.

One surprising finding is the non-significant OR value of age for reversible and potentially reversible cognitive frailty. Although we found significant age differences in the comparisons of cognitive and physical profiles (Table 1), these affected the cognitively impaired (moderate cognitive impairment) and the physical frail groups, and no differences were observed between the other levels of the cognitive continuum (CU, SCD, and MCI) or between the physically robust and pre-frail groups. In the comparison between reversible and potentially reversible cognitive frail profiles, no differences were observed in relation to age, possibly because moderate cognitively impaired participants, who were almost all frail, were excluded because they did not meet the cognitive frailty criteria (SCD or MCI). The OR of Social support was also not statistically significant, possibly because all of the groups had a mean level of social support higher than the mean level for the Portuguese population, i.e., 64.04 (95% CI 59.22–68.85; Fachado et al., 2007). This finding is consistent with that reported by Navarro-Pardo et al. (2020) who did not find a significant effect of social support (MOSS-SS) in a Spanish sample, and it may be a consequence of the generally high level of family and social support given to older adults in Portugal and Spain.

Our findings on the differences between cognitive frailty profiles in mental health, (such as greater probability of clinical non-psychotic psychiatric disorders in frail than in pre-frail phenotypes, together with the significant ORs in predicting both reversible and potentially reversible cognitive frailty) highlight the association between mental health and cognitive frailty and the importance of assessing depression and other affective disorders that disturb psychological well-being in the early detection of frailty (Andrew and Rockwood, 2007; Shimada et al., 2016; Soysal et al., 2017; Lin et al., 2020; Navarro-Pardo et al., 2020; Xie et al., 2021). Our findings also highlight the importance of mental health intervention to prevent the onset and progression of CF (Apóstolo et al., 2018).

Regarding the role of cognitive reserve, we compared the results obtained by considering the effect in two different ways. One way was to include years of formal education as the only proxy in the model, as in Models 2 and 4 (Tables 3, 4). The other way was to include the dimensions Education, Working activity and Leisure time (Models 2 and 3, Tables 3, 4). The findings show that the models constructed in both ways yielded good fit indexes and explained an acceptable level of variance. However, the models including the three CR dimensions explained a higher percentage of variance and in the case of Model 3 for potentially reversible cognitive frailty, the specificity and sensitivity were higher than in the corresponding Model 4, which only included Years of formal education. Moreover, in Model 3 Cognitive Reserve Leisure time was significant while in Model 4, Years of education was not a significant factor. Although the protective effect of education (measured as years of formal schooling) has been demonstrated in many studies (Facal et al., 2021) we suggest that in people with relatively low levels of education -as in our sample (mean 6.56 years)- the effect of formal education received in childhood and adolescence may be “diluted” over time, but that the effect of social, family and free-time activities throughout life may be maintained. Therefore, we emphasize that social, cultural, family and free-time activities throughout life constitute important proxies or dimensions in the cognitive reserve model (Stern, 2012) and that these should be considered, together with education and professional activities, to be predictors of cognitive frailty.

Regarding cognitive status, we should point out that 10% of the sample performed below the criteria for MCI compatible with moderate cognitive impairment, even though these participants did not meet the criteria for diagnosis of dementia. Most of these participants were frail (74.1%) or pre-frail (22.2%). These finding reveal that many community-dwelling older adults have not received adequate cognitive or physical assessment that allows early detection of cognitive decline and physical frailty in primary care centers as a way of secondary prevention (Ruiz et al., 2020).

The main contribution of this study is the measurement for first time of the prevalence of the reversible (14%) and potentially reversible cognitive frailty (15.2%) in a sample of community-dwelling old adults in Portugal and the identification of mental health and cognitive reserve, mainly in the dimension composed by social, cultural, family and free-time activities throughout life, as predictors of the pre-frail and frail phenotypes combined with Subjective cognitive decline and Mild cognitive impairment. For assessment of cognitive frailty, we strongly recommend mental health screening and evaluation of cognitive reserve, not only by measuring years of formal education, but also by using tools that provide a multidimensional view and a measure of this important construct. Intervention in psychological well-being and lifestyles that positively promote these predictors throughout life in adulthood are also recommended. The strong social support provided by family and community relationships in our sample highlights the importance of increasing vigilance of these indicators in frail people as a preventive measure.

While our study provides some novel insights, some limitations must be acknowledged. The greatest limitation, which has already been assumed, is the convenience sample that should be expanded in the future. Although our sample was balanced in terms of the distribution of age groups, it consisted predominantly of women (71.6%) and we could not verify statistical differences due to sex that were fund in other studies (Roppolo et al., 2017; Navarro-Pardo et al., 2020; Rivan et al., 2020). Given that leisure activities were important predictors of CF and considering that these activities are very depending on wealth or economic level, we recognize as a limitation not having included a variable on the economic level of the participants, in order to verify its relationship with the CRI-q-Leisure time factor, and its predictive value on CF (Adja et al., 2020; Scherrer and Morley, 2021). Studying the economic level in rural vs. urban environments could also overcome that. We assessed cognitive status through MoCA test that is a reliable instrument for diagnosis of MCI, however a more complete cognitive evaluation could be done. In short, we acknowledge that this study reports an approach that should be developed further, while trying to overcome these limitations in a cross-sectional design with a small sample, i.e., by examining a larger sample in a longitudinal study.

## Data availability statement

The raw data supporting the conclusions of this article will be made available by the authors, without undue reservation.

## Ethics statement

The studies involving human participants were reviewed and approved by Comissão de Ética da Santa Casa da Misericórdia do Porto, Portugal. The patients/participants provided their written informed consent to participate in this study.

## Author contributions

PG, DF, MC-M, AP, and OJ-R: study design and writing and revising the manuscript. PG: collecting data. PG, AP, and OJ-R: analysis interpretation of data. All authors contributed to the article and approved the submitted version.

## Funding

This work was financially supported by ERDF funds through the National Research Agency (Spanish Ministry of Science, Innovation and Universities; Projects Ref. PSI2017-89389-C2-1-R and PID2020-114521RB-C21) and the Galician Government [GRC (GI-1807-USC); Ref: ED431-2017/27; ED431C-2021/04]; all with ERDF/FEDER funds.

## Conflict of interest

The authors declare that the research was conducted in the absence of any commercial or financial relationships that could be construed as a potential conflict of interest.

## Publisher’s note

All claims expressed in this article are solely those of the authors and do not necessarily represent those of their affiliated organizations, or those of the publisher, the editors and the reviewers. Any product that may be evaluated in this article, or claim that may be made by its manufacturer, is not guaranteed or endorsed by the publisher.
